# SPIDER: constructing cell-type-specific protein–protein interaction networks

**DOI:** 10.1093/bioadv/vbae130

**Published:** 2024-08-30

**Authors:** Yael Kupershmidt, Simon Kasif, Roded Sharan

**Affiliations:** Blavatnik School of Computer Science, Tel Aviv University, Tel Aviv 69978, Israel; Department of Biomedical Engineering, Boston University, Boston, MA 02215, United States; Blavatnik School of Computer Science, Tel Aviv University, Tel Aviv 69978, Israel

## Abstract

**Motivation:**

Protein–protein interactions (PPIs) play essential roles in the buildup of cellular machinery and provide the skeleton for cellular signaling. However, these biochemical roles are context dependent and interactions may change across cell type, time, and space. In contrast, PPI detection assays are run in a single condition that may not even be an endogenous condition of the organism, resulting in static networks that do not reflect full cellular complexity. Thus, there is a need for computational methods to predict cell-type-specific interactions.

**Results:**

Here we present SPIDER (Supervised Protein Interaction DEtectoR), a graph attention-based model for predicting cell-type-specific PPI networks. In contrast to previous attempts at this problem, which were unsupervised in nature, our model’s training is guided by experimentally measured cell-type-specific networks, enhancing its performance. We evaluate our method using experimental data of cell-type-specific networks from both humans and mice, and show that it outperforms current approaches by a large margin. We further demonstrate the ability of our method to generalize the predictions to datasets of tissues lacking prior PPI experimental data. We leverage the networks predicted by the model to facilitate the identification of tissue-specific disease genes.

**Availability and implementation:**

Our code and data are available at https://github.com/Kuper994/SPIDER.

## 1 Introduction

Protein–protein interaction (PPI) networks are the workhorse behind molecular processes in an organism’s cell. Understanding these networks can assist in the prediction of protein function, elucidation of protein complexes, identification of disease pathways, and drug target prioritization (Sharan *et al.* 2007, Safari-Alighiarloo *et al.* 2014, Gordon *et al.* 2020). However, cellular conditions greatly affect protein function, abundance, and, ultimately, protein interaction. Thus, PPIs are context-specific and depend on the tissue or cell type in question (Segal *et al.* 2004, Bossi and Lehner 2009). This context-dependence is not reflected in public databases as most experimental data to date come from artificial systems that measure the interactions in some arbitrary conditions. Indeed, profiling interactions in a specific cell type could be challenging due to the high number of proteins, the variability of their biochemical properties, the high rate of transient interactions, and the low abundance of some proteins (Huttlin *et al.* 2015). Due to these difficulties, very scarce cell-type specific data are available, calling for computational inference of such interactions.

While there is a growing number of works that leverage deep learning to predict PPIs (see, e.g. Szymborski and Emad 2022, Syrlybaeva and Strauch 2023, Ghosh and Mitra 2024), only few works aim at contextualizing them. Early work in this domain has primarily focused on creating biological context networks (Rachlin *et al.* 2006, Schaefer *et al.* 2013). To this end, for a given context, the general (non-context-specific) PPI network is filtered to include only the proteins associated with that context, typically determined through gene ontology (GO) annotations. Subsequently, various methods have been developed to construct tissue-specific networks. One such method, node removal (NR) (cf. Magger *et al.* 2012), involves removing nodes from the network that are not expressed in a given tissue (see, e.g. Ziv *et al.* 2022). A more refined, edge reweighting (ERW) method scores each interaction by the product of the probabilities that its nodes are expressed in the tissue in question (Magger *et al.* 2012). Another approach introduced in Laman Trip *et al.* (2024) infers tissue-specific associations in an unsupervised manner based on protein co-abundance information. To calibrate the association scores, the authors rely on protein complex annotations from the CORUM collection (Ruepp *et al.* 2010). Less systematic approaches are based on text mining but as a result are affected by literature biases (see, e.g. Federico and Monti 2021).

A related body of work relies on tissue-specific networks to conduct downstream prediction tasks. Ohmnet (Zitnik and Leskovec 2017) predicts the cellular function of a protein using a multi-layer network that reflects the known tissue hierarchy. Each layer in the network represents a tissue-specific network that is built using an NR-like strategy of maintaining only nodes that represent tissue-specific or ubiquitous genes. GIANT (Greene *et al.* 2015) utilizes NR-like tissue-specific networks and data from genome-wide association studies to construct tissue-specific functional networks. The recent PINNACLE model uses tissue-specific networks to learn tissue-specific protein representations (Li *et al.* 2023). Those networks are again built by maintaining genes with higher-than-average expression, compared to other cell types or tissues.

Recent experimental advances have allowed for the first time the direct measurement of tissue-specific interactions. Specifically, the BioPlex network project (Huttlin *et al.* 2015) measures two human cell-type-specific networks in two cell lines: HEK293T and HCT116. Additionally, seven mouse tissue networks were measured by Skinnider *et al.* (2021). The availability of experimentally curated tissue- and cell-type-specific networks provides a solid foundation for the development of condition-specific network predictors, facilitating the potential curation of new contexts.

In this work, we introduce SPIDER (Supervised Protein Interaction DEtectoR), a graph attention-based framework for constructing condition-specific PPI networks. SPIDER utilizes diverse protein and interaction features to extract interaction patterns within the dataset that are smoothed using the graph attention component. We showcase the model’s proficiency in capturing interaction characteristics and predicting tissue-specific interactions among a set of proteins based on their features. We demonstrate the superiority of these networks over earlier works in both humans and mice. We further demonstrate the model’s capability to generalize predictions to datasets describing cell types or tissues the model has not been trained on. Finally, we show the application of SPIDER to construct tissue-specific networks and cancer-type-specific networks across a host of human tissues and their utility in identifying disease genes. Overall, the SPIDER model is shown to be a robust and efficient method for computationally inferring context-specific networks with applications to downstream classification tasks.

## 2 Methods

**Figure 1.**
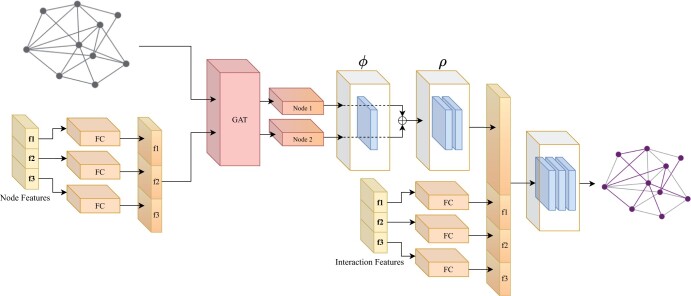
SPIDER’s neural network architecture. Node features, including gene expression, protein expression, and protein localization, as well as interaction features, including protein co-expression, protein co-localization, and interaction discovery techniques, are projected into a joint space using a feed-forward neural network (FFNN) component with two fully connected (FC) layers. Node features are further processed with a graph attention (GAT) layer and a deepest component which represents a ϕ function with one FC layer and a ρ function with two FC layers. The final FFNN component consists of three FC layers and outputs the interaction’s probability.

We developed a deep learning framework for inferring a PPI network in a condition of interest. Our model receives as input two types of information: (i) general information that includes a set of potential interactions, derived from experimental measurements in arbitrary conditions, their detection techniques and their proteins’ cellular location; and (ii) condition-specific information on gene expression and protein abundance. These gene-level and interaction-level features are fed into a neural network for their integration and subsequent use for prediction of condition-specific interactions. The model is trained using real experimental data of PPIs that were directly measured in the specified condition. The model’s architecture is sketched in Fig. 1. Further details regarding the data and methodology are outlined below.

### 2.1 PPI network data

We used PPI information from both mouse and human. For mouse, seven tissue-specific networks were taken from Skinnider *et al.* (2021). These tissue-specific networks varied in size, spanning a range of 20 000–35 000 interactions and involving 1300–2000 proteins. A general (non-context-specific) network was produced by taking the union of these networks and the mouse BioGRID network (Oughtred *et al.* 2018), resulting in 202 443 interactions among 11 795 proteins.

For human, a general network was sourced from BioGRID (Oughtred *et al.* 2018), spanning 1 030 456 physical interactions involving 19 609 proteins. Cell-type specific networks for HEK293T and HCT116 were taken from the Bioplex project (Huttlin *et al.* 2021). The HEK293T network spans 118 162 interactions between 13 957 proteins; the HCT116 network includes 70 966 interactions among 10 115 proteins. The general network covers about 89% of both HEK293T and HCT116 interactions. Additionally, a text mining-based network for HeLa was taken from Federico and Monti (2021) and includes 253 506 interactions (87% of which are covered by the general network) among 16 423 proteins. As acknowledged by the authors of Federico and Monti (2021), their text mining approach may extract interactions that do not occur under the specified conditions, thus we expect such interactions to have lower reliability than experimentally based ones. For this reason, we used only the HeLa network which was the largest network in Federico and Monti (2021).

Henceforth, we focus on interactions that are included in the general BioGRID network. As PPI datasets are known to suffer from high false positive rates (Peng *et al.* 2017), information regarding the experimental methods used for interaction discovery was obtained from BioGRID and used for reliability estimation. For every interaction, we recorded the assays by which it was discovered and the number of times it was discovered in each. The technique features included affinity chromatography technology, anti-bait coimmunoprecipitation, anti-tag coimmunoprecipitation, two-hybrid, pull down, transcriptional complementation assay, affinity technology, biochemical, enzymatic study, proximity labeling technology, and a final category grouping all other experimental interaction detection methods. These features were used to characterize human interactions only, as a large portion of the mouse interactions were only reported in Skinnider *et al.* (2021).

### 2.2 Gene and protein expression data

Human gene expression data, measured in each of the three cell types, were sourced from the Cancer Cell Line Encyclopedia (Ghandi *et al.* 2019). The data include ${\;\text{log}\;}_{2}$(TPM + 1) values, which were normalized to a range between 0 and 1. For the mouse networks, the gene expression data were extracted from Noguchi *et al.* (2017). These expression values underwent log transformation to align with the human gene expression features and were subsequently normalized to values between 0 and 1.

Protein abundance data for HEK293T and HCT116 cells were taken from the Bioplex project (Huttlin *et al.* 2015). Each cell line had five samples, with the protein abundance values computed by the median across those samples. Protein abundance data for HeLa cells were sourced from Itzhak *et al.* (2017). Each protein was measured across 30 samples and the protein value was calculated as the median of the sample-specific values. Protein abundance information for the mouse tissues was taken from Skinnider *et al.* (2021). Protein abundance values for both human and mouse were normalized to be between 0 and 1. Co-abundance values were computed by Pearson correlation of the sample-specific values.

### 2.3 Cellular localization

Cellular location information was derived from the GO annotation. For each protein, a vector was constructed, describing its probability of being localized in a predefined set of primary compartments within the cell (63 for human and 62 for mouse). These compartments were delineated based on terms employed in the LOCATE database (Fink *et al.* 2006). The probability of a protein being localized to a compartment was considered to be uniformly distributed across all GO cellular compartments it is associated with from the specified set. The co-localization of protein pairs was computed by the Pearson correlation between their localization vectors.

### 2.4 Neural network architecture

The SPIDER architecture consists of several layers (Fig. 1). The first layer projects the various input features into a joint space. This is accomplished by passing each feature, such as gene expression, protein abundance, and co-abundance, through a feed-forward neural network (FFNN) component. Next, a graph attention layer aggregates information on each protein from its network neighbors, as described in Brody *et al.* (2021). Subsequently, for each interaction, we utilize a deep set function to merge the embeddings of its two proteins using an order-invariant function. As shown in Zaheer *et al.* (2017), such a function can be approximated by a neural network architecture where each element of the pair is passed through a fully connected layer ϕ, the outputs are summed, and the result passes through another fully connected layer ρ. The resulting embedding is concatenated with data pertaining to the interaction itself, including the co-localization of the proteins, their co-abundance and information regarding the assays used to detect the interaction. These features are then processed through a feed-forward component, ultimately producing the probability of the interaction being a part of the cell-type-specific network.

### 2.5 Model training and hyperparameter tuning

The network models were trained for 750 epochs using an Adam optimizer with a weight decay of 0.01 and a learning rate of 0.001 (see hyperparameter tuning process below). The loss function employed was binary cross-entropy, computed as the difference between the predictions of each interaction and its true label determined by the gold standard context-specific network. For the training and testing process, the nodes of the general network were categorized into three groups: train, validation, and test, of sizes 70%, 15%, and 15%, respectively. The graphs induced by those node sets defined the corresponding edge train, validation, and test sets.

The model’s hyperparameters include the embedding size for the representation of proteins, the learning rate, the dropout probability of the feed-forward components, and the number of training iterations. The dimensions of the feed-forward layers were determined based on the proteins’ embedding size. A range of hyperparameter values was explored, including embedding sizes of [32, 64, 128], learning rates of [1e−3,5e−4,1e−4], dropout probabilities of [0.2, 0.3, 0.4], and iteration numbers of [500, 750, 1000]. Given the data imbalance, the combination yielding the highest AUPRC score on the validation set was selected. The tuning was conducted using the HEK293T dataset as the most extensively researched and experimentally curated network out of the 10 human and mouse datasets. This optimal configuration consisted of an embedding size of 64, a learning rate of 0.001, a dropout probability of 0.3, and 750 epochs.

### 2.6 Baseline methods

We compared our classification results against three baseline methods: NR, ERW (cf. Magger *et al.* 2012) and co-abundance (Laman Trip *et al.* 2024). In NR, the condition-specific network is constructed by projecting gene expression onto the network nodes. Only nodes representing expressed genes are retained in the network, while nodes corresponding to non-expressed genes are removed along with the edges connected to them. The cutoff for a gene to be considered expressed can vary. In ERW, the weights of the edges are modified according to the expression of the genes comprising it. The algorithm sets a penalty factor *rw*, and modifies each edge weight $w_{i,j}$ by multiplying it by $rw^{n}$ where *n* ranges from 0 to 2 and represents the number of lowly or unexpressed genes among the interacting genes. Following Magger *et al.* (2012), we used a penalty factor of rw=0.001. The AUROC (area under the receiver operating characteristic)and AUPRC (area under the precision–recall curve) scores for both methods were computed by varying the gene expression threshold. In co-abundance, the association between a pair of proteins is computed based on the Pearson correlation between their expression values across samples. These scores are further refined using a logistic model that is based on protein complex information from CORUM as explained in Laman Trip *et al.* (2024).

## 3 Results

We developed SPIDER, a graph attention-based model for inferring cell-type-specific PPIs. The predictions employ both protein-level features, such as protein expression and location, and interaction-level features, such as detection assays. In the following, we describe the performance of the model, its comparison to previous work and its application to predicting tissue-specific networks and disease genes.

### 3.1 Performance evaluation

**Figure 2.**
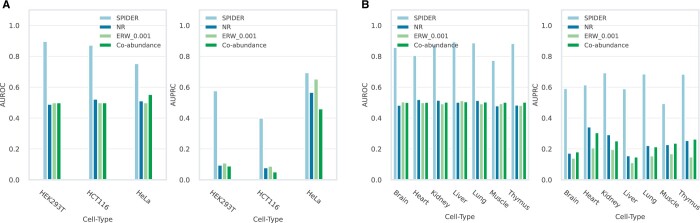
AUROC and AUPRC scores for the human (A) and mouse (B) models and comparison to previous approaches.

We trained 10 cell-type specific models using gold standard networks of various cell types, including three human cell types (HEK293T, HCT116, and HeLa) and seven mouse tissues (brain, heart, kidney, liver, lung, muscle and thymus). We assessed the performance of the models in cross-validation by measuring the AUROC and AUPRC (Fig. 2). Each classifier was trained using both context-specific datasets including gene expression and protein abundance information, as well as, non-context-specific ones including protein location and interaction detection information.

For the human data, the HEK293T and HCT116 models received AUROCs of 0.9 and 0.86, respectively, while the HeLa model achieved an AUROC of 0.77. The relatively lower value for HeLa may reflect the fact that the HeLa ground truth network was not experimentally measured and its text mining construction may be biased. Furthermore, the three models performed well w.r.t. the AUPRC measure, yielding a score of 0.63 for the HEK293T model, 0.34 for the HCT116 model, and 0.71 for the HeLa model, substantially higher than the rate of positive interactions in those datasets (12%, 6%, and 30%, respectively). Similarly, good results were obtained for the mouse data with an average AUROC of 0.85 and AUPRC of 0.62.

We compared our performance to three unsupervised baselines: NR, ERW, and co-abundance (see Methods for details). One potential advantage of SPIDER over previous unsupervised methods w.r.t. the human dataset is the reliance on information regarding the experimental evidence for each interaction, which allows it to focus on more reliable interactions. To remove this bias, we performed the comparison when restricting attention to a subset of reliable interactions. To this end, we applied the pipeline in Signorini *et al.* (2021) to score interactions and for each method set a threshold that maximized the AUROC score obtained. The results are portrayed in Fig. 2 and show the superiority of SPIDER over the three baselines across all cell types.

### 3.2 Generalization and transfer learning

Next, we evaluated SPIDER’s capacity to generalize predictions to new cell types or tissues that the model has not been trained on. To this end, we applied each of the models trained on a specific cell type to predict the other cell types (within the same species). The generalization results are presented in Table 1.

**Table 1.** Generalization AUROC scores of the different human (a) and mouse (b) models.

(a)	Dataset		
Model	HEK293T	HCT116	HeLa
HEK293T	(–)	0.85	0.54
HCT116	0.85	(–)	0.56
HeLa	0.69	0.57	(–)

(b)	Dataset						
Model	Brain	Heart	Kidney	Liver	Lung	Muscle	Thymus
Brain	(–)	0.72	0.82	0.87	0.85	0.77	0.86
Heart	0.78	(–)	0.83	0.87	0.85	0.76	0.81
Kidney	0.81	0.75	(–)	0.89	0.87	0.75	0.86
Liver	0.83	0.76	0.86	(–)	0.88	0.76	0.86
Lung	0.81	0.75	0.85	0.88	(–)	0.76	0.88
Muscle	0.74	0.67	0.76	0.79	0.78	(–)	0.82
Thymus	0.82	0.76	0.85	0.89	0.88	0.76	(–)

For human, both HEK293T and HCT116 models demonstrate the ability to predict one another network nearly as effectively as their original models. However, as shown in Table 1, neither the HEK293T model nor the HCT116 model generalizes well to HeLa. This may reflect the fact that the latter network was deduced from text mining, exposing it to potential literature biases, while the other two networks were systematically explored through experimentation. For mouse, the results reveal that each mouse tissue-specific network can be predicted with high accuracy by any of the other tissue-specific models, with an average AUROC of 0.81. In some cases, such as the predictions of the kidney model on the liver dataset, the generalized model performs as well as the original model. In the case of the brain model predictions on the muscle dataset, the generalized model outperforms the original muscle-trained model. This suggests promising generalization capabilities of the models across different cellular contexts within the same species.

**Figure 3.**
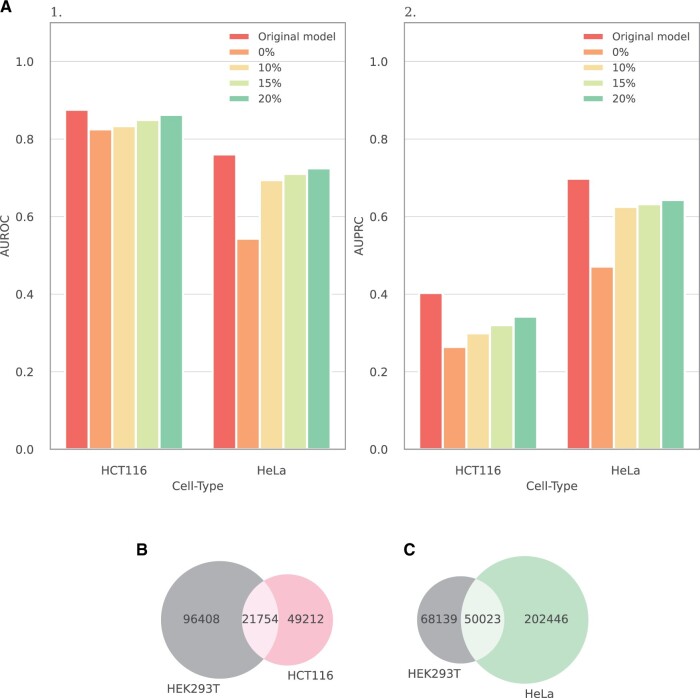
(A) AUROC (A.1) and AUPRC (A.2) scores for the transfer learning from the HEK293T model using varying portions of training data from a target cell (HCT116 or HeLa). The left bar in each panel corresponds to the original model that was trained on the target cell. The 0% bar represents the performance of the HEK293T model w.r.t. other cell types without further training. (B, C) Venn diagrams displaying the intersection of the HEK293T network with the two other human networks.

In an effort to enhance the human network predictions, we pursued the fine-tuning of the HEK293T model, which had the most comprehensive experimental dataset, in order to represent other cell types, leveraging transfer learning techniques. Specifically, after the original training of the HEK293T model, the weights of the initial layers of the model that process the protein-level features were frozen. Thereafter, the model underwent additional training on datasets of different sizes from either HCT116 or HeLa cell types. To prevent overfitting on relatively small training sets, the fine-tuning was executed with a low learning rate of 5e−4 and limited to only 100 training epochs. Figure 3 illustrates the score improvement achieved through the fine-tuning of the HEK293T model with growing fractions of data from the target cell type. The improvement in both AUROC and AUPRC is evident already after introducing the HEK293T model with 10% of the dataset. This is particularly notable given the small fraction of edges that the HEK293T network covers in the two other networks (31% of HCT116 and 20% of HeLa, see Fig. 3B and C), underscoring the model’s generalization power.

### 3.3 Building tissue-specific networks

As a next step, we constructed tissue-specific networks using transcriptomic and proteomic data curated by the Genotype-Tissue Expression (GTEx) project (Jiang *et al.* 2020). This dataset encompasses gene and protein expression from 32 human tissues with multiple samples per tissue.

In order to apply SPIDER to these data, we trained a human predictive model using both the HEK293T and HCT116 datasets. The HeLa dataset was excluded due to its non-experimental assembly. This model underwent training and validation using the combined train and validation sets of the two cell lines, respectively. Its performance was evaluated on the test sets of the two cell lines, achieving an AUROC score of 0.89 and an AUPRC of 0.57 on the HEK293T test set, and an AUROC score of 0.87 and an AUPRC of 0.33 on the HCT116 test sets, closely resembling the scores of the original cell line models.

Next, we applied the combined model to the GTEx data to construct the tissue-specific networks. Since each of the GTEx tissues had only a few samples (2–10) and their gene expression values displayed low variation (mean coefficient of variation per gene in the range 0.2–0.49), we constructed one network per tissue by utilizing the median gene- and protein-expression values to compute interaction classification features. The median expression values underwent the same normalization process as the cell line data. We also created an unweighted version of the networks by setting a threshold on the probabilities of the edges output by the model. This threshold was set to 0.39, as the one that maximizes the combined model’s *F*1-score on the HEK293T and HCT116 test sets.

In order to validate the constructed networks, we sought to compare their pairwise similarities to those implied by the curated Brenda tissue ontology (Gremse *et al.* 2010). Following Li *et al.* (2023), we computed a pairwise tissue ontology distance by the sum of path lengths between the two tissue nodes to their lowest common ancestor. We correlated these distances with the corresponding distances between the (weighted) tissue-specific networks. The distance between a pair of networks was computed using Pearson distance (defined as 1−r, where *r* denotes the Pearson correlation). As a benchmark, we also computed this correlation for tissue-specific networks constructed according to the NR method, where the gene expression threshold was chosen as the value maximizing the *F*_1_-score on the combined HEK293T and HCT116 dataset. The correlation obtained by the SPIDER networks was 0.23 (P<1.52e−5), while the other method led to a close-to-zero negative correlation of −0.02.

**Figure 4.**
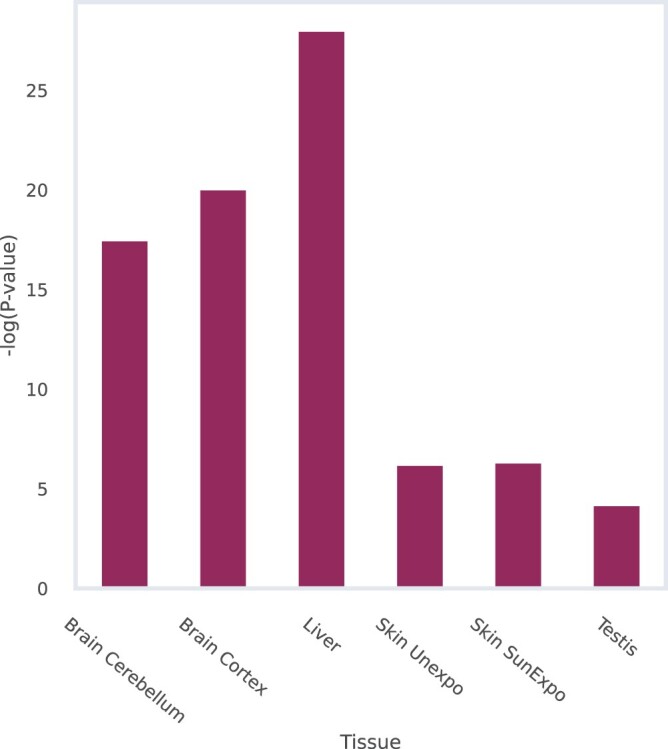
Significance values (−log(*p*-value)) of a Wilcoxon rank-sum test between degree differences of tissue-specific proteins and all other proteins, where (relative) degree differences are computed between the general network degree and a tissue-specific network degree.

As another validation of the tissue-specific networks, we checked if the interactions within them are enriched with proteins that are known to be specifically expressed in the corresponding tissues. To this end, we utilized the tissue-enriched gene sets defined by The Human Protein Atlas (Uhlen *et al.* 2010), focusing on tissues with at least 25 annotated proteins. For each protein, we computed its relative degree (i.e. degree percentile) change between any tissue-specific network (unweighted version) and the general network. We then compared the distribution of these values in a given tissue between the tissue-specific proteins and all other proteins using a Wilcoxon rank-sum test. The results are portrayed in Fig. 4 and, reassuringly, all tissues display significant differences.

**Figure 5.**
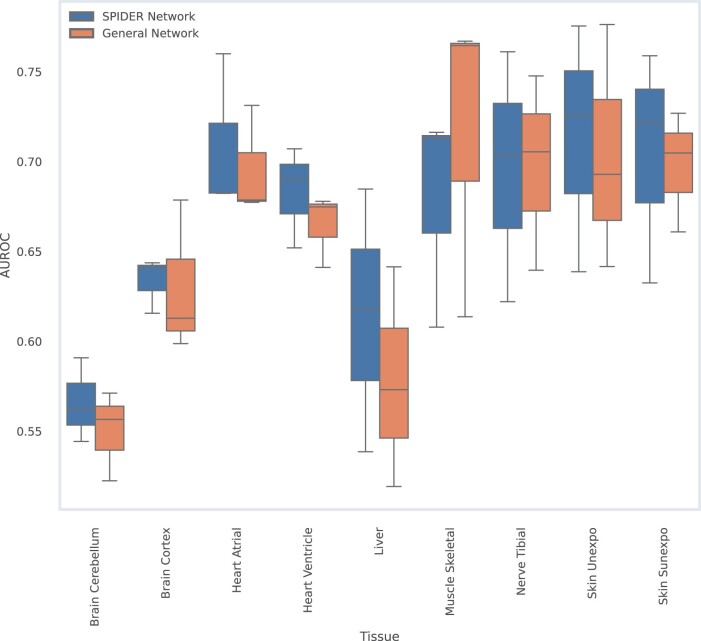
AUROC box plots for tissue-associated disease gene prediction.

To further validate the tissue-specific networks, we tested their utility at pinpointing tissue-specific disease genes. To this end, we used a dataset reported in Simonovsky *et al.* (2023) of 1 117 tissue-associated disease genes affecting 23 tissues. In order to predict tissue-specific genes, we used network propagation (Cowen *et al.* 2017) in a 3-fold cross-validation setting. That is, each fold was predicted based on propagating from a seed composed of the genes from the other two folds. For the propagation process, we used the network’s adjacency matrix normalized by vertex degrees (stochastic normalization) and a default 0.8 value for the α parameter that describes the tradeoff between network smoothing and prior information. To remove genes for which no interaction prediction could be made, each tissue-specific gene group was filtered to include only genes measured by GTEx in the respective tissue. Gene sets with <25 genes were discarded. Figure 5 presents the resulting AUROC scores, comparing them to predictions made using the general network.

Evidently, the tissue-specific networks exhibit a notable advantage in predicting tissue-specific disease genes.

### 3.4 Identifying cancer-driver genes

Last, we tested the application of the model in constructing a cancer-type-specific network and utilizing it for the identification of cancer-driver genes. To this end, three datasets from the Cancer Genome Atlas (TCGA) were incorporated (breast cancer, colon adenocarcinoma, and ovarian cancer). The selection of these datasets was based on the availability of protein expression for at least 30% of the proteins in the network.

Unlike the GTEx data, TCGA datasets included hundreds of samples with high gene expression variation (mean coefficient of variation of 1.6–2.8). Thus, we generated an individual network for each sample within a cancer type, employing the HEK293T and HCT116 combined model and the sample’s features. As there are no multiple measurements per sample, the co-abundance feature was computed using the sample’s protein abundance and the protein abundance measurements of the five samples that are most similar to it (measured via the cosine similarity of the samples’ protein expression vectors). Then, we aggregated the sample-specific networks to form a “consensus network” for each cancer type, where the weight of each edge in the consensus network was determined as the mean edge weight across all sample-specific networks.

**Figure 6.**
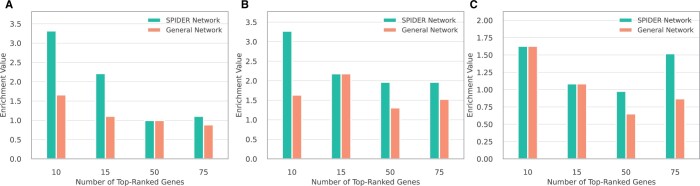
Enrichment values w.r.t. cancer-associated genes of top *K* predictions in breast cancer (A), colon adenocarcinoma (B), and ovarian cancer (C) cancer-specific networks.

Finally, we used the consensus network to predict cancer-driver genes. For each sample, we propagated its mutated gene set in the corresponding consensus network to score all other genes. The mutated gene set included genes that were reported to be mutated or to exhibit copy number alterations. These predictions were then averaged over all samples of a specific cancer type. A gold standard set of driver genes was defined by the COSMIC Cancer Gene Census (CGC) (Sondka *et al.* 2018). Figure 6 presents the enrichment of the CGC genes within the top-ranked genes. For any *K*, the top-*K* enrichment was calculated according to:

(1) $$\frac{n/K}{|\text{CGC}|/N},$$

where *n* is the number of CGC genes within the *K* top-ranked genes, and *N* denotes number of proteins in the consensus network. Evidently, the predictions made by the sample-specific networks dominate those made with a general network across all three cancer types.

## 4 Conclusions

PPI networks are the workhorse of the cell but can vary substantially between cell types and tissues. In this study, we have successfully developed and trained a predictive model for cell-type-specific PPIs. Our model integrates gene expression information, protein abundance data, protein location information, and interaction detection assay data, and demonstrates improvements on multiple evaluation fronts and benchmarks as compared to previous methods in this space.

One of the advantages of using advanced machine learning models of this type is their ability to generalize to novel cell types where relevant experimental data have been collected without doing full scale PPI studies which might be cost- or labor-prohibitive. Indeed, we show that our model can generalize to yet-unseen tissues or cell types. Additionally, we implemented a transfer learning strategy to fine-tune such predictions and demonstrated that even with a small training set the model can be refined effectively to predict the target network with sufficient accuracy. Finally, we showcased the utility of the framework in predicting tissue-specific disease genes and cancer-driver genes. SPIDER can thus serve as a useful resource for downstream tissue-specific classification tasks.

While SPIDER has shown good performance, several of its limitations should be acknowledged. First, condition-specific features could be enhanced depending on the availability of such data. Second, training and validation are limited by the relatively small number of condition-specific networks, particularly in humans. With the accumulation of condition-specific data, future work could investigate how networks vary between different conditions, providing insights into the dynamic nature of PPIs and their roles in different biological contexts.

## Data Availability

The datasets used in this study were sourced from publicly available repositories. The general networks for both human and mouse were obtained from BioGRID (https://downloads.thebiogrid.org/BioGRID). The HEK293T and HCT116 cell-line-specific networks, along with protein expression data, were provided by the Bioplex project and are accessible at https://bioplex.hms.harvard.edu/interactions.php. All mouse tissue-specific networks and associated protein expression data are published in https://doi.org/10.1016/j.cell.2021.06.003. Human gene expression data were derived from the CCLE dataset (https://sites.broadinstitute.org/ccle/datasets), while mouse gene expression data were obtained from the FANTOM5 dataset (https://fantom.gsc.riken.jp/data/). Protein expression data for the HeLa cell-line can be found at https://doi.org/10.1016/j.celrep.2017.08.063. Localization data were sourced from the Gene Ontology and are available for download at https://geneontology.org/docs/download-ontology. The gene and protein expression data for the human tissue-specific networks were obtained from https://doi.org/10.1016/j.cell.2020.08.036, and the corresponding data for cancer-type-specific networks were retrieved from the TCGA project, accessible at https://www.cbioportal.org/datasets. The tissue-enriched gene set is published by the Human Protein Atlas (https://www.proteinatlas.org/about/download), and tissue-specific disease genes are available at https://doi.org/10.15252/msb.202211407.

## References

[vbae130-B1] Bossi A , Lehner B. Tissue specificity and the human protein interaction network. Mol Syst Biol 2009;5:260. 19357639 10.1038/msb.2009.17 PMC2683721

[vbae130-B2] Brody S , Alon U, Yahav E. How attentive are graph attention networks? arXiv, arXiv:2105.14491, 2021, preprint: not peer reviewed.

[vbae130-B3] Cowen L , Ideker T, Raphael BJ et al Network propagation: a universal amplifier of genetic associations. Nat Rev Genet 2017;18:551–62. 28607512 10.1038/nrg.2017.38

[vbae130-B4] Federico A , Monti S. Contextualized protein–protein interactions. Patterns (N Y) 2021;2:100153. 33511361 10.1016/j.patter.2020.100153 PMC7815950

[vbae130-B5] Fink JL , Aturaliya RN, Davis MJ et al Locate: a mouse protein subcellular localization database. Nucleic Acids Res 2006;34:D213–7. 16381849 10.1093/nar/gkj069 PMC1347432

[vbae130-B6] Ghandi M , Huang FW, Jané-Valbuena J et al Next-generation characterization of the cancer cell line encyclopedia. Nature 2019;569:503–8. 31068700 10.1038/s41586-019-1186-3 PMC6697103

[vbae130-B7] Ghosh S , Mitra P. MaTPIP: a deep-learning architecture with explainable AI for sequence-driven, feature mixed protein–protein interaction prediction. Comput Methods Programs Biomed 2024;244:107955. 38064959 10.1016/j.cmpb.2023.107955

[vbae130-B8] Gordon DE , Jang GM, Bouhaddou M et al A SARS-CoV-2 protein interaction map reveals targets for drug repurposing. Nature 2020;583:459–68. 32353859 10.1038/s41586-020-2286-9 PMC7431030

[vbae130-B9] Greene CS , Krishnan A, Wong AK et al Understanding multicellular function and disease with human tissue-specific networks. Nat Genet 2015;47:569–76. 25915600 10.1038/ng.3259 PMC4828725

[vbae130-B10] Gremse M , Chang A, Schomburg I et al The BRENDA tissue ontology (BTO): the first all-integrating ontology of all organisms for enzyme sources. Nucleic Acids Res 2010;39:D507–13. 21030441 10.1093/nar/gkq968 PMC3013802

[vbae130-B11] Huttlin EL , Bruckner RJ, Navarrete-Perea J et al Dual proteome-scale networks reveal cell-specific remodeling of the human interactome. Cell 2021;184:3022–40.e28. 33961781 10.1016/j.cell.2021.04.011 PMC8165030

[vbae130-B12] Huttlin EL , Ting L, Bruckner RJ et al The BioPlex network: a systematic exploration of the human interactome. Cell 2015;162:425–40. 26186194 10.1016/j.cell.2015.06.043 PMC4617211

[vbae130-B13] Itzhak DN , Davies C, Tyanova S et al A mass spectrometry-based approach for mapping protein subcellular localization reveals the spatial proteome of mouse primary neurons. Cell Rep 2017;20:2706–18. 28903049 10.1016/j.celrep.2017.08.063 PMC5775508

[vbae130-B14] Jiang L , Wang M, Lin S et al; GTEx Consortium. A quantitative proteome map of the human body. Cell 2020;183:269–83.e19. 32916130 10.1016/j.cell.2020.08.036 PMC7575058

[vbae130-B15] Laman Trip DS , van Oostrum M, Memon D et al An atlas of protein–protein associations of human tissues prioritizes candidate disease genes. bioRxiv, 2024, preprint: not peer reviewed. 10.1101/2024.05.15.594301

[vbae130-B16] Li MM , Huang Y, Sumathipala M et al Contextualizing protein representations using deep learning on protein networks and single-cell data. bioRxiv, 2023, preprint: not peer reviewed. 10.1101/2023.07.18.549602.

[vbae130-B17] Magger O , Waldman YY, Ruppin E et al Enhancing the prioritization of disease-causing genes through tissue specific protein interaction networks. PLoS Comput Biol 2012;8:e1002690. 23028288 10.1371/journal.pcbi.1002690 PMC3459874

[vbae130-B18] Noguchi S , Arakawa T, Fukuda S et al FANTOM5 CAGE profiles of human and mouse samples. Sci Data 2017;4:170112. 28850106 10.1038/sdata.2017.112 PMC5574368

[vbae130-B19] Oughtred R , Stark C, Breitkreutz B-J et al The BioGRID interaction database: 2019 update. Nucleic Acids Res 2018;47:D529–41. 10.1093/nar/gky1079 PMC6324058 30476227

[vbae130-B20] Peng X , Wang J, Peng W et al Protein–protein interactions: detection, reliability assessment and applications. Brief Bioinform 2017;18:798–819. 27444371 10.1093/bib/bbw066

[vbae130-B21] Rachlin J , Cohen DD, Cantor C et al Biological context networks: a mosaic view of the interactome. Mol Syst Biol 2006;2:66. 17130868 10.1038/msb4100103 PMC1693461

[vbae130-B22] Ruepp A , Waegele B, Lechner M et al CORUM: the comprehensive resource of mammalian protein complexes—2009. Nucleic Acids Res 2010;38:D497–501. 19884131 10.1093/nar/gkp914 PMC2808912

[vbae130-B23] Safari-Alighiarloo N , Taghizadeh M, Rezaei-Tavirani M et al Protein–protein interaction networks (PPI) and complex diseases. Gastroenterol Hepatol Bed Bench 2014;7:17–31. 25436094 PMC4017556

[vbae130-B24] Schaefer MH , Lopes TJS, Mah N et al Adding protein context to the human protein–protein interaction network to reveal meaningful interactions. PLoS Comput Biol 2013;9:e1002860. 23300433 10.1371/journal.pcbi.1002860 PMC3536619

[vbae130-B25] Segal E , FriedmanN, KollerD et al A module map showing conditional activity of expression modules in cancer. Nat Genet2004;36:1090–8.15448693 10.1038/ng1434

[vbae130-B26] Sharan R , Ulitsky I, Shamir R et al Network-based prediction of protein function. Mol Syst Biol 2007;3:88. 17353930 10.1038/msb4100129 PMC1847944

[vbae130-B27] Signorini LF , Almozlino T, Sharan R et al ANAT 3.0: a framework for elucidating functional protein subnetworks using graph-theoretic and machine learning approaches. BMC Bioinformatics 2021;22:526. 34706638 10.1186/s12859-021-04449-1 PMC8555137

[vbae130-B28] Simonovsky E , Sharon M, Ziv M et al; GTEx Consortium. Predicting molecular mechanisms of hereditary diseases by using their tissue-selective manifestation. Mol Syst Biol 2023;19:e11407. 37232043 10.15252/msb.202211407 PMC10407743

[vbae130-B29] Skinnider MA , Scott NE, Prudova A et al An atlas of protein–protein interactions across mouse tissues. Cell 2021;184:4073–89.e17. 34214469 10.1016/j.cell.2021.06.003

[vbae130-B30] Sondka Z , Bamford S, Cole CG et al The cosmic cancer gene census: describing genetic dysfunction across all human cancers. Nat Rev Cancer 2018;18:696–705. 30293088 10.1038/s41568-018-0060-1 PMC6450507

[vbae130-B31] Syrlybaeva R , Strauch E-M. Deep learning of protein sequence design of protein–protein interactions. Bioinformatics 2023;39:btac733. 36377772 10.1093/bioinformatics/btac733 PMC9947925

[vbae130-B32] Szymborski J , Emad A. RAPPPID: towards generalizable protein interaction prediction with AWD–LSTM twin networks. Bioinformatics 2022;38:3958–67. 35771595 10.1093/bioinformatics/btac429

[vbae130-B33] Uhlen M , Oksvold P, Fagerberg L et al Towards a knowledge-based human protein atlas. Nat Biotechnol 2010;28:1248–50. 21139605 10.1038/nbt1210-1248

[vbae130-B34] Zaheer M , Kottur S, Ravanbakhsh S et al Deep sets. In: *Advances in Neural Information Processing Systems*, 2017, 3394–404.

[vbae130-B35] Zitnik M , Leskovec J. Predicting multicellular function through multi-layer tissue networks. Bioinformatics 2017;33:i190–8. 28881986 10.1093/bioinformatics/btx252 PMC5870717

[vbae130-B36] Ziv M , Gruber G, Sharon M et al The TissueNet v.3 database: protein–protein interactions in adult and embryonic human tissue contexts. J Mol Biol 2022;434:167532. 35662455 10.1016/j.jmb.2022.167532

